# Thermodynamic profiling during irreversible electroporation in porcine liver and pancreas: a case study series

**Published:** 2020-03-12

**Authors:** Pierre Agnass, Eran van Veldhuisen, Jantien A. Vogel, H. Petra Kok, Mark J. de Keijzer, Gerben Schooneveldt, Lianne R. de Haan, John H. Klaessens, Hester J. Scheffer, Martijn R. Meijerink, Krijn P. van Lienden, Thomas M. van Gulik, Michal Heger, Johannes Crezee, Marc G. Besselink

**Affiliations:** ^1^Department of Surgery, Cancer Center Amsterdam, Amsterdam UMC, University of Amsterdam, Amsterdam, The Netherlands; ^2^Department of Radiation Oncology, Cancer Center Amsterdam, Amsterdam UMC, University of Amsterdam, Amsterdam, The Netherlands; ^3^Department of Experimental Surgery, Amsterdam UMC, University of Amsterdam, Amsterdam, The Netherlands; ^4^Department of Pharmaceutics, Utrecht Institute for Pharmaceutical Sciences, Utrecht University, Utrecht, The Netherlands; ^5^Department of Clinical Physics, Medical Center Leeuwarden, Leeuwarden, The Netherlands; ^6^Department of Radiology and Nuclear Medicine, Cancer Center Amsterdam, Amsterdam UMC, Vrije Universiteit Amsterdam, Amsterdam, The Netherlands; ^7^Department of Radiology, Cancer Center Amsterdam, Amsterdam UMC, University of Amsterdam, Amsterdam, The Netherlands; ^8^Department of Pharmaceutics, College of Medicine, Jiaxing University, Jiaxing, Zhejiang, P.R. China

**Keywords:** complications, experimental pig model, irreversible electroporation, microwave ablation, mild hyperthermia, numerical treatment planning, radiofrequency ablation, therapy, tissue ablation

## Abstract

**Aims:**

First, the aim of the study was to determine whether irreversible electroporation (IRE) is associated with heat generation in the liver and pancreas at clinical (≤1,500 V/cm) and supraclinical (>1,500 V/cm) electroporation settings; second, to assess the risk of thermal tissue damage in and adjacent to the treated volume in highly perfused versus moderately perfused parts of both organs; third, to investigate the influence of perfusion and of the presence and the orientation of a metal stent on the maximal thermal elevation (ΔT_Session,max_) in the tissue during an IRE session at fixed IRE settings, and finally, to determine whether the maximum temperature elevation within the IRE-subjected organ during an IRE treatment (single or multiple sessions) is reflected in the organ’s surface temperature.

**Methods:**

The aims were investigated in 12 case studies conducted in five female Landrace pigs. Several IRE settings were applied for lateral (2), triangular (3), and rectangular (4) electrode configurations in the liver hilum, liver periphery, pancreas head, and pancreas tail. IRE series of 10-90 pulses were applied with pulse durations that varied from 70 μs to 90 μs and electric field strengths between 1,200 V/cm and 3,000 V/cm. In select cases, a metal stent was positioned in the bile duct at the level of the liver hilum. Temperatures were measured before, during, and after IRE in and adjacent to the treatment volumes using fiber optical temperature probes (temperature at the nucleation centers) and digital thermography (surface temperature). The occurrence of thermal damage was assumed to be at temperatures above 50 °C (ΔT_Session,max_ ≥ 13 °C relative to body temperature of 37 °C). The temperature fluctuations at the organ surface (ΔT_LocSurf_) were compared to the maximum temperature elevation during an IRE treatment in the electroporation zone. In select cases, IRE was applied to tissue volumes encompassing the portal vein (PV) and a constricted and patent superior mesenteric vein (SMV) to determine the influence of the heatsink effect of PV and SMV on ΔT_Session,max_.

**Results:**

The median baseline temperature was 31.6 °C-36.3 °C. ΔT_Session,max_ ranged from –1.7 °C to 25.5 °C in moderately perfused parts of the liver and pancreas, and from 0.0 °C to 5.8 °C in highly perfused parts. The median ΔT_LocSurf_ of the liver and pancreas was 1.0 °C and 10.3 °C, respectively. Constricting the SMV in the pancreas head yielded a 0.8 °C higher ΔT_Session,max_. The presence of a metal stent in the liver hilum resulted in a ΔT_Session,max_ of 19.8 °C. Stents parallel to the electrodes caused a ΔT_Session,max_ difference of 4.2 °C relative to the perpendicular orientation.

**Conclusions:**

Depending on IRE settings and tissue type, IRE is capable of inducing considerable heating in the liver and pancreas that is sufficient to cause thermal tissue damage. More significant temperature elevations are positively correlated with increasing number of electrode pairs, electric field strength, and pulse number. Temperature elevations can be further exacerbated by the presence and orientation of metal stents. Temperature elevations at the nucleation centers are not always reflected in the organ’s surface temperature. Heat sink effects caused by large vessels were minimal in some instances, possibly due to reduced blood flow caused by anesthesia.

**Relevance for patients:**

Appropriate IRE settings must be chosen based on the tissue type and the presence of stents to avoid thermal damage in healthy peritumoral tissue and to protect anatomical structures

**Table:** 

Abbreviation and definitions	Unit	Description
IRE		Irreversible electroporation
IRE session		Application of IRE with fixed IRE settings
IRE treatment		A treatment that includes a single or multiple IRE sessions. In case of multiple sessions, IRE settings were varied
NA		Not applicable
TP		Fiber optical temperature probe
T	[°C]	Temperature
T_LocSurf,init_	[°C]	Initial surface temperature obtained from a local area on the treated organ
T_LocSurf,max_	[°C]	Maximal surface temperature obtained from a local area on the treated organ
T_Surf_	[°C]	Surface temperature of the IRE-subjected organ measured by a digital thermography
T_TP,init_	[°C]	Initial temperature in or near the IRE-subjected volume measured by a single fiber optical temperature probe in the tissue before IRE
T_TP,max_	[°C]	Maximal temperature in or near the treated volume measured by a single fiber optical temperature probe in the tissue
ΔT_Session,max_	[°C]	Maximally measured thermal elevation during an IRE session (the maximal difference between the maximal temperature during and after IRE, and the initial temperature before the start of IRE in a single IRE session)
ΔT_LocSurf_	[°C]	The difference between the maximal and the initial localized surface temperature measured by digital thermography
ΔT_TP_	[°C]	The difference between the maximal and the initial temperature measured by a single fiber optical temperature probe in the tissue in a single session
ΔT_Treat,max_	[°C]	The difference between the maximal and the minimal temperature measured by a single fiber optical temperature probe in the tissue during an IRE treatment

## 1. Introduction

Irreversible electroporation (IRE) is an image-guided focal ablation technique that is currently investigated in clinical trials for the treatment of patients with unresectable tumors, including but not limited to a variety of pancreatic cancers, centrally located liver tumors, and urological cancers [1-5]. With IRE, high-voltage, sub-millisecond electrical pulses are applied across electrode pairs, which are placed in and around a tumor. The electrical pulses perturb the cell membrane potential [6], leading to the disruption of the lipid bilayer, after which the cell loses homeostatic control and dies by mainly apoptosis [7]. Cancer cells that undergo necrosis release damage-associated molecular patterns and tumor-associated antigens that mediate sterile inflammation and, depending on the downstream signaling cascades, an anti-tumor immune response, respectively [8,9].

Preclinical studies have shown that IRE only affects cells in the direct zone of ablation, sparing collagenous structures and thereby preserving the tissue scaffolding [10,11]. Preservation of the gross anatomic architecture allows safe ablation of tumors near vital structure such as blood vessels. Theoretically, the non-thermal mechanism of cell death implies that heat sink effects should not impede treatment efficacy [12]. For those reasons, IRE is becoming increasingly popular as an alternative to thermal ablation techniques for intervening at locations where thermal damage is of concern.

Although IRE was initially introduced as a non-thermal technique, the application of repetitive high-intensity electrical pulses in the liver, pancreas, and kidney inevitably leads to heating that may result in thermal damage when a thermal threshold (50 °C-60 °C) is reached [13-16]. In particular, this might be the case due to the presence of metal implants, such as metal clips, staples, and stents. Specifically, in the direct vicinity of the metal implants the electric field distribution can be distorted, and the deposition of thermal energy can increase due to the relative large thermal conductivity of metal with respect to the treated tissue, resulting in possible thermal damage [17,18]. Several studies in pigs have demonstrated that increased temperatures can occur in the liver, pancreas, and kidney during IRE, depending on the IRE parameters [17,19,20]. Still, a limited number of *in vivo* studies determined the extent of tissue heating during IRE, especially in the liver and the pancreas. In addition, to the best of our knowledge, no *in vivo* experiments have been performed in a single study regarding heat sink effects during IRE in the vicinity of large blood vessels such as the portal vein and the superior mesenteric vein, and at multiple less perfused locations. The influence of a metal stent in the bile duct on thermogenesis during IRE, including its orientation, also warranted closer examination.

To determine the extent of heating in the liver and pancreas during IRE, several IRE settings need to be explored in different *in vivo* setups while measuring the real-time temperature in the treatment zone. IRE treatments in these organs are performed in comparatively more highly perfused regions (i.e., in the vicinity of the portal vein in the liver hilum and the superior mesenteric vein in the pancreatic head) and in more moderately perfused regions (i.e., in the liver periphery and pancreatic tail). The organs are thus heterogeneously perfused and potentially prone to more effective cooling due to convection. Hemodynamic heat removal may have a favorable dampening effect on temperature evolution and consequently limit the degree of thermal damage. At the same time, differences may arise between locoregional temperatures and the surface temperature, which may lead to inaccurate temperature read-outs during perioperative thermal profiling and faulty clinical decision-making.

Accordingly, in this case series study, we addressed the following research questions: (1) Does the maximal temperature elevation (ΔT_Session,max_, the greatest difference between the maximal temperature during and after IRE, and the initial temperature before the start of IRE in a single IRE session) potentially cause thermal tissue damage in the liver and pancreas; (2) does the heat sink effect in the highly perfused parts of these organs significantly influence ΔT_Session,max_; (3) are temperature elevations within the IRE-subjected organ reflected in the organ’s surface temperature; and (4) does the presence and the orientation of a metal stent influence the ΔT_Session,max_? Periprocedural temperature measurements were used to gauge potential thermal tissue damage. Temperature readouts constitute a continuous parameter to quantitatively assess the risk of thermal injury compared to histological analysis, which is dichotomous, semi-quantitative at best, and non-paired.

## 2. Animals and Methods

Following approval by the institutional animal ethics committee (protocol BEX102047), consecutive IRE ablations were performed in the liver and pancreas of five female Landrace pigs (±50 kg) with and without a metal biliary stent.

### 2.1. Animal procedure

The animals were anesthetized with intramuscular ketamine (10-15 mg/kg), midazolam (1-1.5 mg/kg), and atropine (0.03 mg/kg and 1.5 mL/50 kg). After intubation, anesthesia was maintained through inhaled isoflurane (2%-4%) in air (20% oxygen) and intravenous ketamine (2 mg/kg/h), sufentanil (5-10 mg/kg/h), midazolam (1-2 mg/kg/h), and rocuronium bromide (2-2.5 mg/kg/h). Before IRE, intravenous bolus injections of rocuronium (1-1.5 mg/kg) were administered for complete muscle relaxation. The animals were placed in supine position and the liver and pancreas were mobilized following a medial laparotomy. During IRE the animals were kept in apnea to minimize ventilatory motion. The animals were euthanized by exsanguination within 30 min after the final ablation.

### 2.2. IRE procedure

The IRE procedures were conducted as case series (*n* = 1 per unique experiment), where multiple cases were performed in one animal in line with the Dutch guidelines for animal research (strive for reduction in the number of animals used). For optimal legibility and understandability, the experimental detail for each case study is presented in the Results section.

**Figure 1:**
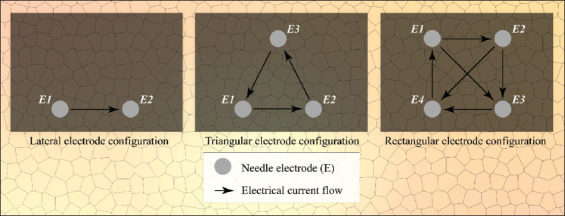
Electrode configurations used in this study, including electrical current flow directions. Please note that during IRE treatment, the electrode pairs were used successively and not concurrently.

IRE was performed with a Nanoknife system (AngioDynamics, Latham, NY) in the liver hilum, liver periphery, pancreas head, and pancreas tail. Depending on the experiment, the number of applied electrode pairs varied from 2 to 6 with an active tip length of 1.5-2.0 cm, inter-electrode distance of 1.0-2.0 cm, and electrode configurations, as depicted in Figure 1. Furthermore, series of 10-90 pulses were applied in 1-5 sessions with pulse durations that varied from 70 μs to 90 μs, and electric field strengths between 1,200 V/cm and 3,000 V/cm. These parameters are used in the clinical setting for electric field strengths of 1,200 V/cm-1,500 V/cm. The reason for including supraclinical settings (>1,500 V/cm) was to obtain electrical currents of >20 A in a select number of cases, which is the minimum required current to ablate tissue according to the protocol of AngioDynamics and Martin [21]. When the electrical current exceeds the safety threshold of 50 A, the Nanoknife automatically aborts the IRE procedure.

In case of the liver hilum, IRE experiments were performed with and without a nitinol stent (Boston Scientific, Marlborough, MA). The stents were placed under ultrasound guidance at a liver depth of approximately 1.0-1.5 cm parallel to the surface of the liver. The stents were expanded *in situ* to a diameter of 0.5 cm. Electrodes were positioned parallel and perpendicular to the stent without the use of ECHO guidance.

### 2.3. Temperature measurements

To measure the temperature (T) during IRE within the IRE-subjected organ, up to three fiber optical temperature probes (TP) were placed under ECHO guidance directly within and/or adjacent to the ablation zone at equal depth relative to the IRE electrode tips. The fiber optical temperature setup consisted of a rack-mounted Lumiterm X5 OEM temperature board connected to 3 LumitermX5-True fiber optical temperature probes with a diameter of 1 mm (IPITEK, Carlsbad, CA). The system is capable of registering temperature differences of 0.05 °C with an accuracy of 0.2 °C and is not affected by the presence of an electric field. The temperature-recording interval was standardized to 1 s. For every temperature probe in each session, the initial temperature (T_TP,init_[°C]) before the IRE procedure and the corresponding maximal temperature (T_TP,max_ [°C]) during and after the IRE procedure were measured. The temperature difference (ΔT_TP_ [°C]) was calculated from these values. Please note that ΔT_TP_ represents temperature difference of each probe per session, while ΔT_Session,max_ represents the maximal temperature difference in a single session.

In seven out of 12 experiments, the surface temperature (T_Surf_) of the liver and the pancreas was continuously measured during IRE using a Gobi-384 thermal camera (Xenics, Leuven, Belgium). This camera is calibrated to measure the temperature in a range of −20 °C-120 °C. Furthermore, the camera is capable of registering temperature differences of 0.05 °C at a resolution of 384 × 288 pixels (25-mm pitch). The temperature data were extracted from a case-depended local area using Xeneth Software (Xenics) to determine the initial local T_Surf_ (T_LocSurf,init_ [°C]) before the IRE procedure and the maximal local T_Surf_ (T_LocSurf,max_ [°C]) during and after IRE. The average temperature was determined at sites with the highest T_LocSurf,max_ in the field of view based on the intensity values of 1-5 pixels. Using the option “Color Threshold” in ImageJ (National Institutes of Health, Bethesda, MD), we could determine the average temperature and calculate the difference (ΔT_LocSurf_ [°C]). Furthermore, the ΔT_LocSurf_ was reflected in the ΔT_Session,max_ during an IRE treatment (ΔT_Treat,max_), measured by a single TP inside or outside the treated volume. The ΔT_Treat,max_ was calculated as the maximal difference between the maximal and the minimal temperature per probe during the treatment. In case of multiple TPs inside the treated volume, the maximal value of ΔT_Treat,max_ was selected. As an additional step, the linear regression was calculated to determine the relationship between ΔT_LocSurf_ and ΔT_Treat,max_.

### 2.4. Assessment of thermally ablative temperatures

To estimate whether IRE induces temperature elevations sufficiently high to cause thermal damage, a temperature threshold of 50 °C (ΔT_Session,max_ ≥ 13 °C assuming an initial body temperature of 37 °C) was selected inasmuch as damage to tissue increases significantly beyond this point [16]. Assessment of the risk of thermal damage was performed according to the temperature traces in and near the ablation zone and the thermal images generated with the infrared camera.

## 3. Results

### 3.1. Summated main findings of the case studies

**Figure 2:**
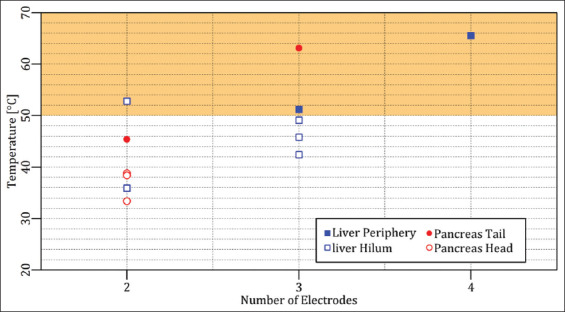
Maximally measured temperature of each case study as a function of the number of electrodes. The orange rectangle delineates the thermal damage zone.

To provide an overview of the case studies, the results of each case are summarized in Table 1 and the maximally measured temperatures were plotted as a function of the selected number of electrodes in Figure 2. Furthermore, the accessibility of the Results section was preserved by moving the figures in cases 2-5 and 10-12 into the Appendix section. figures in the Appendix are indicated with a prefix ‘A’.

**Table 1:** Overview of the case studies. Readers should note that: (1) NA is defined as “Not applicable” and (2) the median baseline temperature was 31.6 °C-36.3 °C.

Case number	Organ	Notes	Electrode number	Number of sessions	Pulse parameters	Maximally measured temperature within IRE-subjected organ [°C] (*ΔT_Treat,max_*)	Maximal surface temperature [°C] (*ΔT_LocSurf_*)	Was the thermal damage threshold reached?
1	Liver periphery	NA	4	3	1,500 V/cm 90 μs 20-90 pulses	65.5 °C (*29.2 °C*)	30.7 °C (*1 °C*)	Yes
2	Liver periphery	NA	3	1	1,500 V/cm 70 μs 90 pulses	51.2 °C (*16.7 °C*)	25 °C (*0 °C*)	Yes
3	Liver hilum	Electrodes parallel to portal vein	3	3	1,500 V/cm 90 μs 20-90 pulses	42.4 °C (*6.1 °C*)	37 °C (*6 °C*)	No
4	Liver hilum	Electrodes parallel to bile duct containing a stent	3	1	1,500 V/cm 70 μs 90 pulses	49.1 °C (*14.6 °C*)	29 °C (*3 °C*)	No
5	Liver hilum	Electrodes perpendicular to bile duct containing a stent	3	1	1,500 V/cm 70 μs 90 pulses	45.8 °C (*10.4 °C*)	28.5 °C (*0 °C*)	No
6	Liver hilum	Electrodes parallel to bile duct, no stent	2	2	1,200-1,500 V/cm 90 μs 50-90 pulses	35.9 °C (*0.2 °C*)	NA	No
7	Liver hilum	Electrodes parallel to bile duct containing a stent	2	2	1,500 V/cm 90 μs 20-70 pulses	52.8 °C (*21.1 °C*)	NA	Yes
8	Pancreas tail	NA	3	5	1,500-3,000 V/cm 90 μs 20-90 pulses	63.1 °C (*30.4 °C*)	37 °C (*13 °C*)	Yes
9	Pancreas tail	NA	2	5	1,500-2200 V/cm 90 μs 20-90 pulses	45.4 °C (*13.6 °C*)	29 °C (*7.5 °C*)	No
10	Pancreas head	Electrodes in vicinity of the superior mesenteric vein	2	3	1,500 V/cm 90 μs 20-90 pulses	33.4 °C (*0.3 °C*)	NA	No
11	Pancreas head	Electrodes surrounding superior mesenteric vein	2	2	1,350-1,500 V/cm 90 μs 10-90 pulses	38.8 °C (*3.4 °C*)	NA	No
12	Pancreas head	Electrodes in vicinity of constricted superior mesenteric vein	2	1	1,350 V/cm 90 μs 90 pulses	38.4 °C (*4.1 °C*)	NA	No

### 3.2. Liver

#### 3.2.1. Case 1: liver periphery – four electrodes

**Figure 3:**
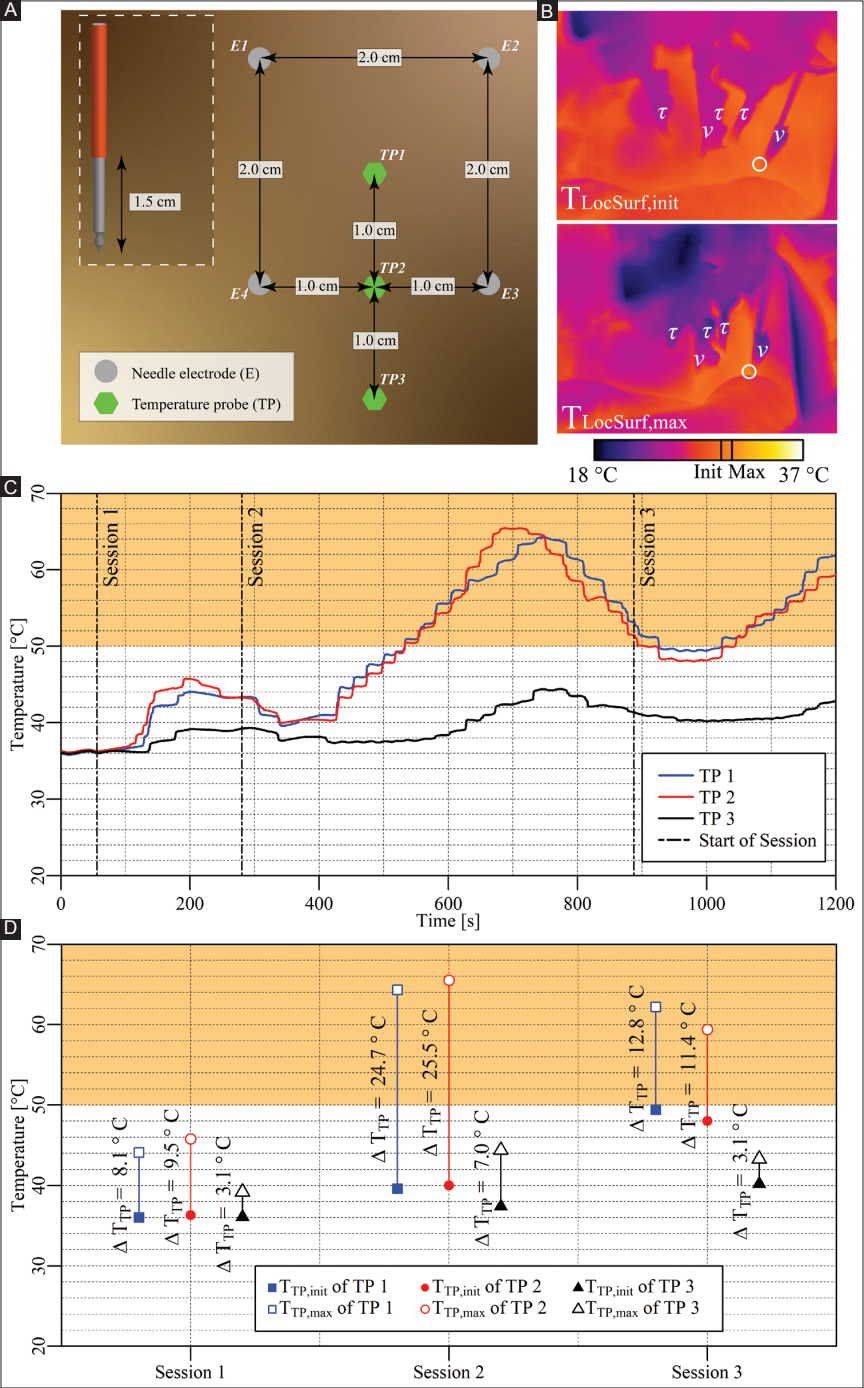
**(A)** Configuration of the electrodes and the temperature probes in the liver periphery (case 1). **(B)** Thermal camera images of the IRE site. The T_LocSurf,init_ (upper panel) and the T_LocSurf,max_ (lower panel) were calculated from pixels within the encircled areas. Electrodes are indicated by “υ” and temperature probes by “τ.” Two electrodes did not appear in these images due to their locations behind the temperature probes. On the color bar, the initial and maximal temperatures are indicated by “Init” and “Max.” **(C)** Tissue temperature profile as a function of time and IRE session. **(D)** T_TP,init_, T_TP,max_, and ΔT_TP_ of each temperature probe in each session, with TP1 and TP2 inside the treated region and TP3 outside the treated region. The orange rectangles in **(C)** and **(D)** indicate the thermal damage zone.

Four electrodes with an active tip length of 1.5 cm were inserted in rectangular configuration in the liver periphery (Figure 3A). Three consecutive IRE sessions were performed with an interval of 4 min between the start of sessions 1 and 2 and an interval of 10 min between the start of sessions 2 and 3. The pulse parameters are listed in Table 2.Figure 3B shows T_LocSurf,max_ ≈ 30.7 °C and ΔT_LocSurf_ ≈ 1 °C. The temperature evolution over time is presented in Figure 3C, provided here as an example and omitted in the rest of the experimental results. On the basis of these traces, the T_TP,init_, T_TP,max_, and ΔT_TP_ of each probe were calculated for every session (Figure 3D). The T_TP,max_ in the ablation zone ranged from 44.1 °C to 65.5 °C (TP1 and TP2), with ΔT_TP_ ranging from 8.1 °C to 25.5 °C. The ΔT_Treat,max_ was 29.2 °C. The maximal temperature exceeded 50 °C for almost 9 min and attests to the manifestation of thermal tissue damage in the IRE-subjected liver periphery. Outside the ablation zone, the T_TP,max_ ranged from 39.2 °C to 44.4 °C (TP3), with ΔT_TP_ ranging from 3.1 °C to 7.0 °C.

**Table 2:** Pulse parameters and electrode pairs (rectangular configuration) of the IRE sessions in the liver periphery (case 1).

Session	Electric field strength [V/cm]	Pulse duration [μs]	Number of pulses	Electrode pair
Session 1	1,500	90	20	1-2
				1-3
				2-3
				2-4
				3-4
				4-1
Session 2	1,500	90	70	1-2
				1-3
				2-3
				2-4
				3-4
				4-1
Session 3	1,500	90	90	1-3
			80*	2-3
			90	2-4

* Due to high current (>50 A), 80 pulses were applied instead of the preprogrammed 90.

#### 3.2.2. Case 2: liver periphery – three electrodes

**Figure A1:**
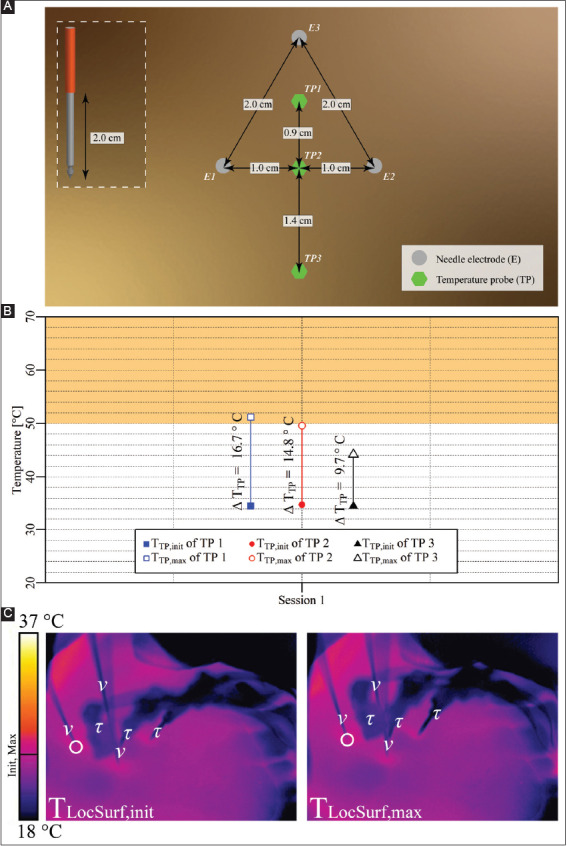
**(A)** Configuration of the electrodes and temperature probes in the liver periphery. **(B)** T_TP,init_, T_TP,max_, and ΔT_TP_ of each probe with TP1 and TP2 inside the treated region and TP3 outside the treated region. The orange rectangle indicates the thermal damage zone. **(C)** Thermal camera images of the IRE site. The T_LocSurf,init_ (left panel) and the T_LocSurf,max_ (right panel) were calculated from pixels within the encircled areas. Electrodes are indicated by “υ” and temperature probes by “τ.” On the color bar, the initial and maximal temperatures are indicated by “Init” and “Max”.

Three electrodes with an active tip length of 2 cm were inserted in triangular configuration in the liver periphery (Figure A1A). A single IRE session was performed with pulse parameters as described in Table 3. The T_TP,init_, T_TP,max_, and ΔT_TP_ of each probe are presented in Figure A1B. The T_TP,max_ in the ablation zone ranged from 49.6 °C to 51.2 °C (TP1 and TP2), with ΔT_TP_ ranging from 14.9 °C to 16.7 °C. The ΔT_Treat,max_ was 16.7 °C. The maximal temperature exceeded 50 °C for 2.1 min. Outside the ablation zone, the T_TP,max_ was 44.2 °C (TP3), with ΔT_TP_ = 9.7 °C.Figure A1C shows T_LocSurf,max_ ≈ 25 °C and ΔT_LocSurf_ ≈ 0 °C.

**Table 3:** Pulse parameters and the electrode pairs (triangular configuration) of the IRE session in the liver periphery (case 2).

Session	Electric field strength [V/cm]	Pulse duration [μs]	Number of pulses	Electrode pair
Session 1	1,500	70	90	1-2
				2-3
				3-1

#### 3.2.3. Case 3: liver hilum – three electrodes parallel to portal vein

**Figure A2:**
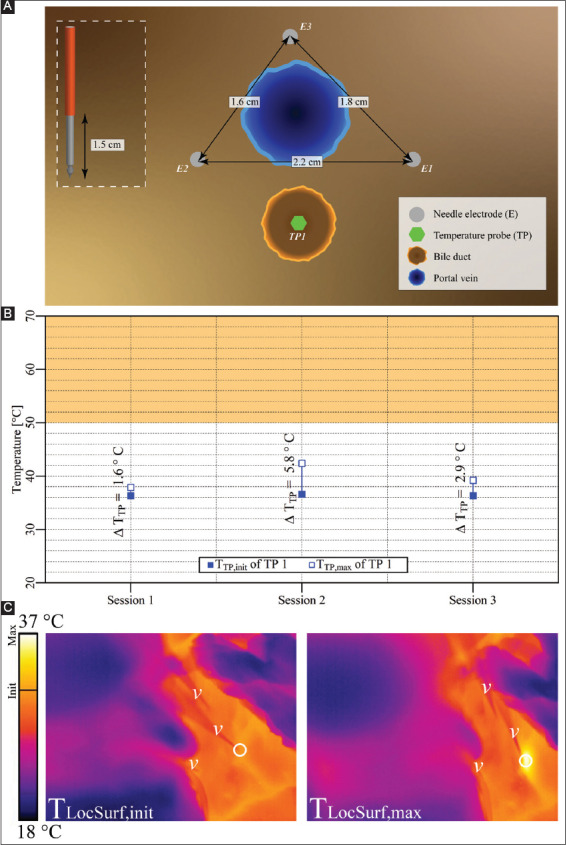
**(A)** Configuration of electrodes and the temperature probe in the liver hilum with electrodes perpendicular to bile duct not containing a stent. **(B)** Measured T_TP,init_, T_TP,max_, and ΔT_TP_ of the temperature probe in each session with TP outside the treated region. The orange rectangle delineates the thermal damage zone. **(C)** Thermal camera images with the T_LocSurf,init_ on the left side and the T_LocSurf,max_ on the right side, extracted from within the white encircled areas. Electrodes are indicated by “υ.” The temperature probe did not appear in these images. On the color bar, the initial and maximal temperatures are indicated by “Init” and “Max”.

Three electrodes with an active tip length of 1.5 cm were inserted in triangular configuration in the liver hilum parallel to the portal vein (Figure A2A). Three consecutive IRE sessions were performed with an interval of 3 min between the start of sessions 1 and 2 and an interval of 7 min between the start of sessions 2 and 3. The pulse parameters are provided in Table 4. The T_TP,init_, T_TP,max_, and ΔT_TP_ for each respective session are presented in Figure A2B. The T_TP,max_ outside the ablation zone ranged from 37.9 °C to 42.4 °C, with ΔT_TP_ ranging from 1.6 °C to 5.7 °C. The ΔT_Treat,max_ was 6.1 °C. Figure A2C shows that T_LocSurf,max_ = 37 °C and ΔT_LocSurf_ ≈ 6 °C.

**Table 4:** Pulse parameters and electrode pairs (triangular configuration) of the IRE sessions in the liver hilum with the portal vein in the treatment region (case 3).

Session	Electric field strength [V/cm]	Pulse duration [μs]	Number of pulses	Electrode pair
Session 1	1,500	90	20	1-2
				3-1
				2-3
Session 2	1,500	90	70	1-2
				3-1
				2-3
Session 3	1,500	90	90	1-2
				3-1
				2-3

#### 3.2.4. Case 4: liver hilum – three electrodes parallel to a bile duct containing a stent

**Figure A3:**
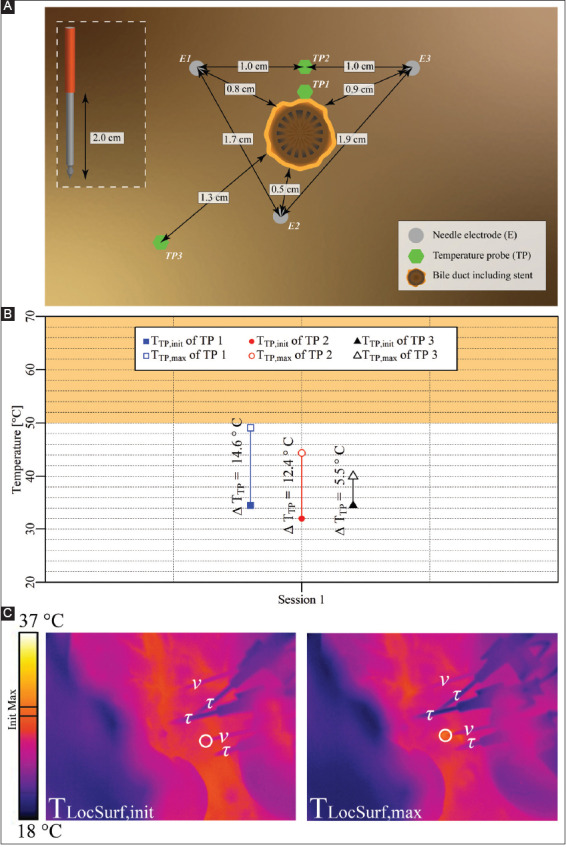
**(A)** Configuration of electrodes and temperature probes in the liver hilum with electrodes parallel to the bile duct containing a stent. **(B)** Measured T_TP,init_, T_TP,max_, and ΔT_TP_ of each probe with TP1 and TP2 inside the treated region, and TP3 outside the treated region. The orange rectangle indicates the thermal damage zone. **(C)** Thermal camera images with the T_LocSurf,init_ on the left side and the T_LocSurf,max_ on the right side, extracted from within the white encircled areas. Electrodes are indicated by “υ” and temperature probes by “τ.” One electrode in both images did not appear due to its location behind the temperature probes. On the color bar, the initial and maximal temperatures are indicated by “Init” and “Max”.

Three electrodes with an active tip length of 2 cm were inserted in triangular configuration in the liver hilum parallel to the bile duct containing a stent, as shown in Figure A3A. A single IRE session was performed with the pulse parameters provided in Table 5. The T_TP,init_, T_TP,max_, and ΔT_TP_ of each probe are shown in Figure A3B. This figure reveals that T_TP,max_ in the ablation zone ranged from 44.4 °C to 49.1 °C (TP1 and TP2), with ΔT_TP_ ranging from 12.4 °C to 14.6 °C. The ΔT_Treat,max_ was 14.6 °C. Outside the ablation zone, T_TP,max_ was 40 °C (TP3), with ΔT_TP_ = 5.5 °C. Figure A3C shows T_LocSurf,max_ ≈ 29 °C and ΔT_LocSurf_ ≈ 3 °C, which considerably deviates from the fiber optic readouts.

**Table 5:** Pulse parameters and electrode pairs (triangular configuration) of the IRE session in the liver hilum and bile duct encompassing a stent in the treatment region (case 4).

Session	Electric field strength [V/cm]	Pulse duration [μs]	Number of pulses	Electrode pair
Session 1	1,500	70	90	2-3
				3-1
				1-2

#### 3.2.5. Case 5: liver hilum – three electrodes perpendicular to a bile duct containing a stent

**Figure A4:**
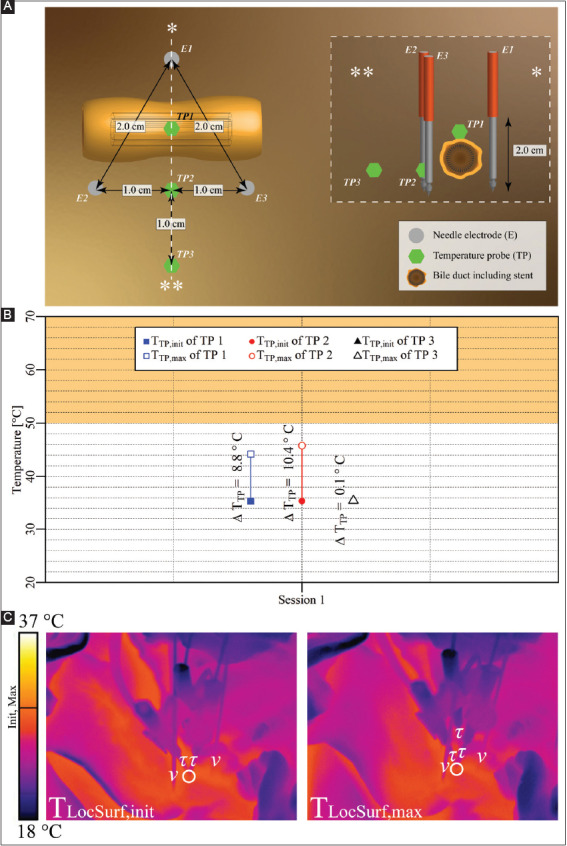
**(A)** Configuration of electrodes and temperature probes in the liver hilum with electrodes perpendicular to bile duct that contained a stent. TP1 was placed on the top of the bile duct, while TP2 and TP3 were placed next to the bile duct. **(B)** Measured T_TP,init_, T_TP,max_, and ΔT_TP_ of each probe in each session with TP1 and TP2 inside the treated region, and TP3 outside the treated region. The orange rectangle indicates the thermal damage zone. **(C)** Thermal camera images with the T_LocSurf,init_ on the left side and the T_LocSurf,max_ on the right side, extracted from within the white encircled areas. Electrodes are indicated by “υ” and temperature probes by “τ”. One electrode in both images and the TP in the left image are not visible due to their locations behind the visible electrodes and temperature probes. On the color bar, the initial and maximal temperatures are indicated by “Init” and “Max”.

Three electrodes with an active tip length of 2 cm were inserted in triangular configuration in the liver hilum perpendicular to a bile duct harboring a stent (Figure A4A). A single IRE session was performed with the pulse parameters provided in Table 6. The T_TP,init_, T_TP,max_, and ΔT_TP_ of each probe are shown in Figure A4B. The figure reveals that T_TP,max_ in the ablation zone ranged from 44.2 °C to 45.8 °C (TP1 and TP2), with ΔT_TP_ ranging from 8.8 °C to 10.4 °C. The ΔT_Treat,max_ was 10.4 °C. Outside the ablation zone, T_TP,max_ was 35.5 °C (TP3), with ΔT_TP_ = 0.1 °C. Figure A4C shows that T_LocSurf,max_ ≈ 28.5 °C and ΔT_LocSurf_ ≈ 0 °C.

**Table 6:** Pulse parameters and electrode pairs (triangular configuration) of the IRE session in the liver hilum with a stent-containing bile duct in the treatment region (case 5).

Session	Electric field strength [V/cm]	Pulse duration [μs]	Number of pulses	Electrode pair
Session 1	1,500	70	90	1-2
				2-3
				3-1

#### 3.2.6. Case 6: liver hilum – two electrodes parallel to a bile duct, no stent

**Figure 4:**
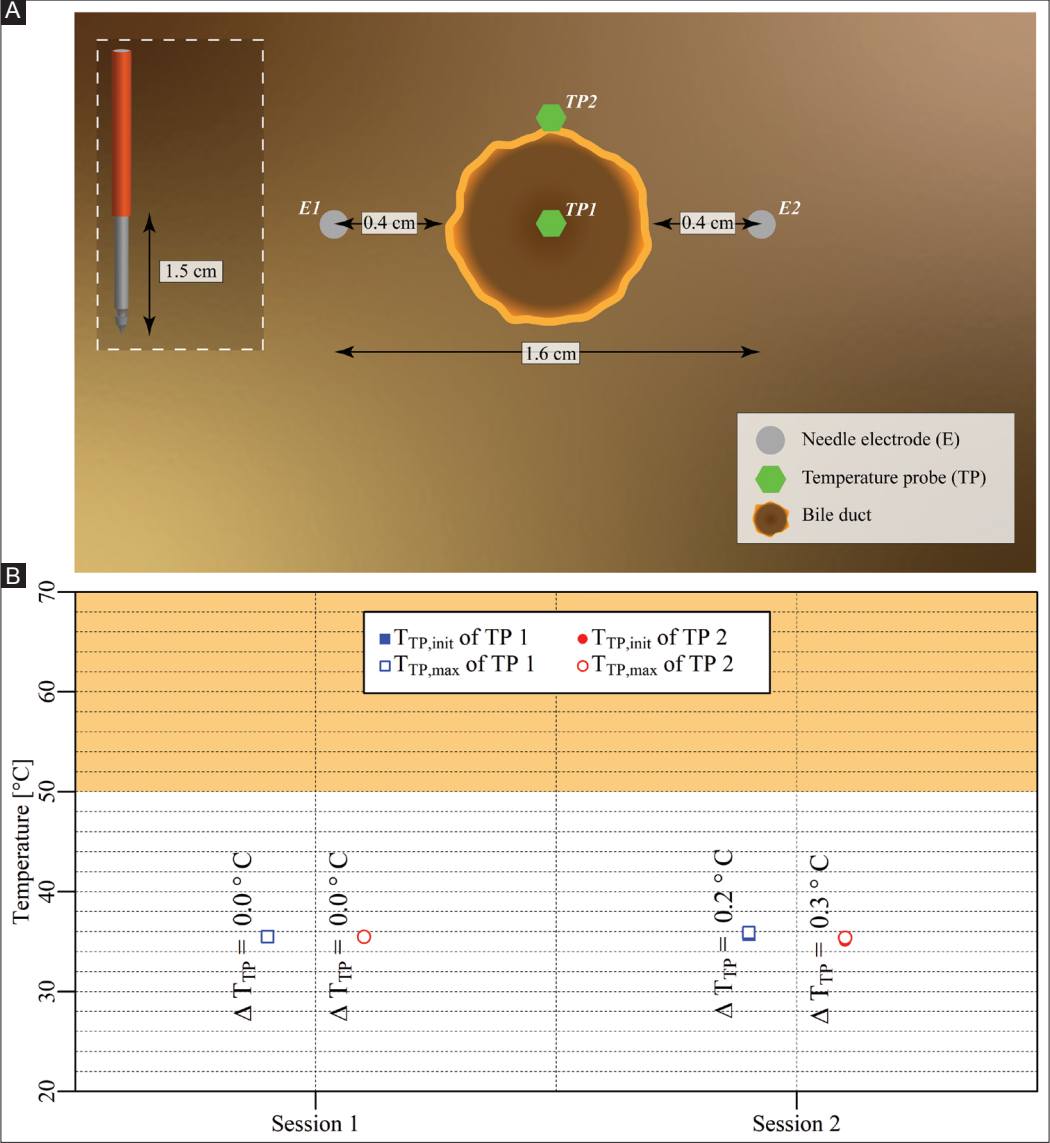
**(A)** Configuration of electrodes and temperature probes in the liver hilum with electrodes parallel to a bile duct not containing a stent (case 6). **(B)** Measured T_TP,init_, T_TP,max_, and ΔT_TP_ of each probe in each session with TP1 and TP2 inside the treated region. The orange rectangle indicates the thermal damage zone.

Two electrodes with an active tip length of 1.5 cm were inserted in the liver hilum parallel to the bile duct, as shown in Figure 4A. Two consecutive IRE sessions were performed with an interval of 1 min. The pulse parameters are provided in Table 7. The T_TP,init_, T_TP,max_, and ΔT_TP_ of each probe for each respective session are shown in Figure 4B. The figure reveals that T_TP,max_ in the ablation zone ranged from 35.4 °C to 35.9 °C (TP1 and TP2), with ΔT_TP_ ranging from 0.0 °C to 0.3 °C. The ΔT_Treat,max_ was 0.3 °C. This case did not entail thermal camera measurements.

**Table 7:** Pulse parameters and electrode pair (lateral configuration) of the IRE sessions in the liver hilum with a bile duct but no stent in the treated region (case 6).

Session	Electric field strength [V/cm]	Pulse duration [μs]	Number of pulses	Electrode pair
Session 1	1,500	90	90	1-2
Session 2	1,200	90	40*	1-2

* Due to high current (>50 A), 40 pulses were applied instead of the preprogrammed 80.

#### 3.2.7. Case 7: Liver hilum – two electrodes parallel to a bile duct containing a stent

**Figure 5:**
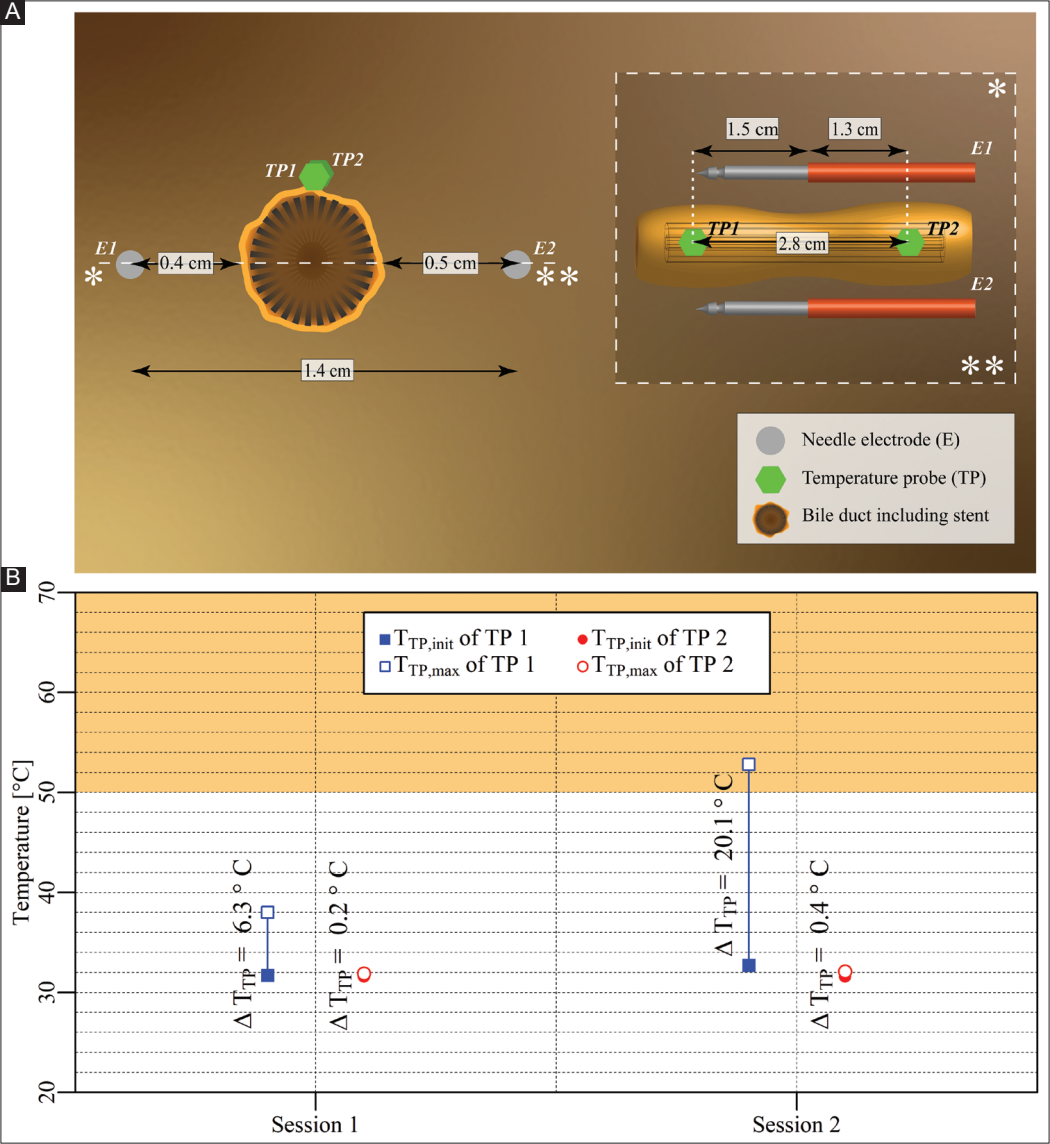
**(A)** Configuration of electrodes and temperature probes in the liver hilum with electrodes parallel to a stent-containing bile duct (case 7). **(B)** Measured T_TP,init_, T_TP,max_, and ΔT_TP_ of each probe in each session with TP1 inside the treated region and TP2 outside the treated region. The orange rectangle designates the thermal damage zone.

Two electrodes with an active tip length of 1.5 cm were inserted in the liver hilum parallel to a stent-containing bile duct, as shown in Figure 5A. The interval between the start of the two IRE sessions was 1 min. The pulse parameters are provided in Table 8. The T_TP,init_, T_TP,max_, and ΔT_TP_ of each probe for each respective session are shown in Figure 5B. This figure reveals that T_TP,max_ in the ablation zone ranged from 31.8 °C to 52.8 °C (TP1 and TP2), with ΔT_TP_ ranging from 0.2 °C to 20.1 °C. The ΔT_Treat,max_ was 21.1 °C. This case did not include thermal camera recordings.

**Table 8:** Pulse parameters and electrode pair (lateral configuration) of the IRE sessions in the liver hilum with a bile duct containing a stent in the treated region (case 7).

Session	Electric field strength [V/cm]	Pulse duration [μs]	Number of pulses	Electrode pair
Session 1	1,500	90	20	1-2
Session 2	1,500	90	70	1-2

### 3.3. Pancreas

#### 3.3.1. Case 8: Pancreas tail – three electrodes

**Figure 6:**
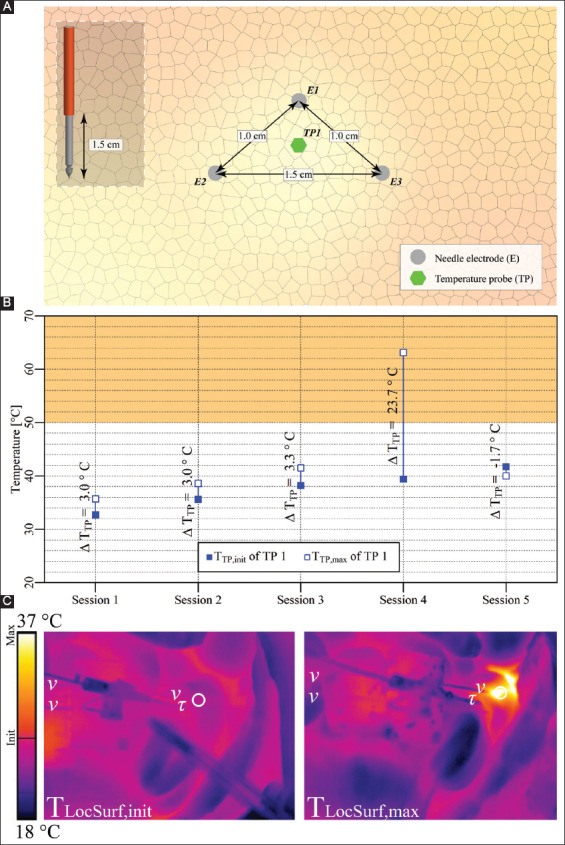
**(A)** Configuration of electrodes and the temperature probe in the pancreas tail (case 8). **(B)** Measured T_TP,init_, T_TP,max_, and ΔT_TP_ of the temperature probe in each session inside the treated region. The orange rectangle indicates the thermal damage zone. **(C)** Thermal camera images with the T_LocSurf,init_ on the left side and the T_LocSurf,max_ on the right side, extracted from within the white encircled areas. Electrodes are indicated by “υ” and temperature probes by “τ.” On the color bar, the initial and maximal temperatures are indicated by “Init” and “Max”.

Three electrodes with an active tip length of 1.5 cm were inserted in triangular configuration in the pancreas tail, as shown in Figure 6A. Five consecutive IRE sessions were performed with an interval of 2.5 min between the start of the first three sessions, and an interval of 11 min between the start of sessions 4 and 5. The pulse parameters are provided in Table 9. The T_TP,init_, T_TP,max_, and ΔT_TP_ of each session are shown in Figure 6B. This figure reveals that T_TP,max_ in the ablation zone ranged from 35.7 °C to 63.1 °C, with ΔT_TP_ ranging from −1.6 °C to 23.7 °C. The ΔT_Treat,max_ was 30.4 °C. The maximal temperature exceeded 50 °C for almost 4.5 min during session 4. Figure 6C shows T_LocSurf,max_ ≈ 37 °C and ΔT_LocSurf_ ≈ 13 °C.

**Table 9:** Pulse parameters and the electrode pairs (triangular configuration) of the IRE sessions in the pancreas tail (case 8).

Session	Electric field strength [V/cm]	Pulse duration [μs]	Number of pulses	Electrode pair
Session 1	1,500	90	20	1-2
				2-3
				3-1
Session 2	1,750	90	20	1-2
				2-3
				3-1
Session 3	2,250	90	20	1-2
				2-3
				3-1
Session 4	3,000	90	90	1-2
	2,000	90	90	2-3
	3,000	90	20*	3-1
Session 5	1,500	90	70	3-1

* Due to high current (>50 A), 20 pulses were applied instead of the preprogrammed 90.

#### 3.3.2. Case 9: Pancreas tail – two electrodes

**Figure 7:**
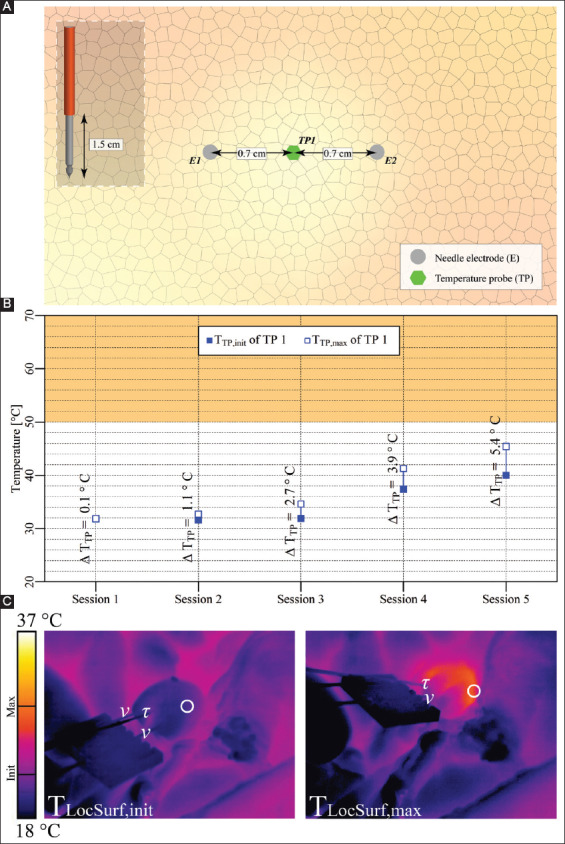
**(A)** Configuration of electrodes and the temperature probe in the pancreas tail (case 9). **(B)** Measured T_TP,init_, T_TP,max_, and ΔT_TP_ of the temperature probe. The orange rectangle indicates the thermal damage zone. **(C)** Thermal camera images with the T_LocSurf,init_ on the left side and the T_LocSurf,max_ on the right side, extracted from within the white encircled areas. Electrodes are indicated by “υ” and temperature probes by “τ.” One electrode in the right image did not appear due to its location behind the temperature probe. On the color bar, the initial and maximal temperatures are indicated by “Init” and “Max”.

Two electrodes with an active tip length of 1.5 cm were inserted in the pancreatic tail, as shown in Figure 7A. Five consecutive IRE sessions were performed with intervals of 2.5 min between the start of the sessions and an interval of 4.5 min between the start of sessions 3 and 4. The pulse parameters are provided in Table 10. The T_TP,init_, T_TP,max_, and ΔT_TP_ of each session are shown in Figure 7B. This figure reveals that T_TP,max_ in the ablation zone ranged from 31.9 °C to 45.4 °C, with ΔT_TP_ ranging from 0.1 °C to 5.4 °C. The ΔT_Treat,max_ was 13.6 °C. Figure 7C shows T_LocSurf,max_ ≈ 29 °C and ΔT_LocSurf_ ≈ 7.5 °C.

**Table 10:** Pulse parameters and electrode pair (lateral configuration) of the IRE sessions in the pancreas tail (case 9).

Session	Electric field strength [V/cm]	Pulse duration [μs]	Number of pulses	Electrode pair
Session 1	1,500	90	20	1-2
Session 2	1,750	90		
Session 3	2,200	90		
Session 4	2,200	90	70	
Session 5	1,800	90	90	

#### 3.3.3. Case 10: Pancreas head – two electrodes in vicinity of the superior mesenteric vein

**Figure A5:**
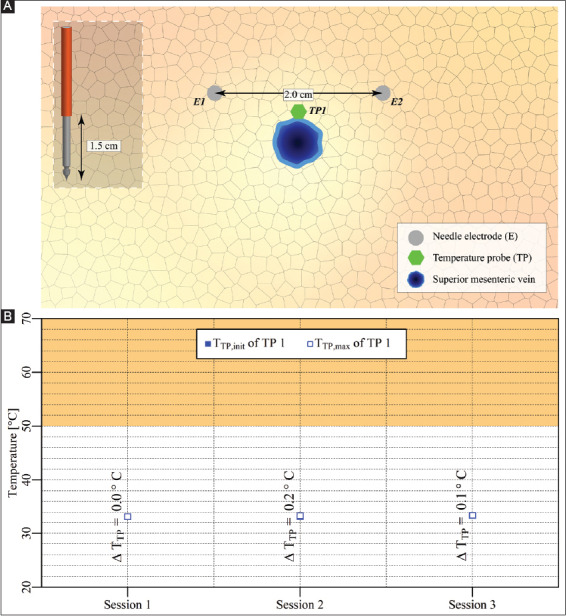
**(A)** Configuration of electrodes and the temperature probe in the pancreas head. **(B)** Measured T_TP,init_, T_TP,max_, and ΔT_TP_ of the temperature probe in each session positioned adjacent to the treated region. The orange rectangle indicates the thermal damage zone.

Two electrodes with an active tip length of 1.5 cm were inserted in the pancreas head above the superior mesenteric vein (Figure A5A). Three consecutive IRE sessions were performed with intervals of 4 min between the start of the sessions. The pulse parameters are provided in Table 11. The T_TP,init,_ T_TP,max_, and ΔT_TP_ of each session are shown in Figure A5B. The figure reveals that T_TP,max_ in the ablation zone ranged from 33.1 °C to 33.4 °C, with ΔT_TP_ ranging from 0 °C to 0.2 °C. The ΔT_Treat,max_ was 0.3 °C. This case did not entail thermal camera measurements.

**Table 11:** Pulse parameters and electrode pair (lateral configuration) of the IRE sessions in the pancreas head with superior mesenteric vein near the treatment region (case 10).

Session	Electric field strength [V/cm]	Pulse duration [μs]	Number of pulses	Electrode pair
Session 1	1,500	90	20	1-2
Session 2	1,500	90	70	1-2
Session 3	1,500	90	90	1-2

#### 3.3.4. Case 11: Pancreas head – two electrodes surrounding the superior mesenteric vein

**Figure A6:**
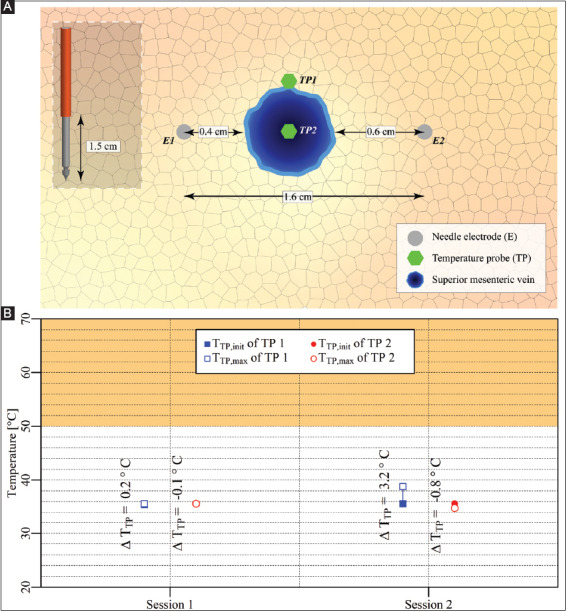
**(A)** Configuration of electrodes and optical fiber temperature probes in the pancreas head. **(B)** Measured T_TP,init_, T_TP,max_, and ΔT_TP_ of each temperature probe in each session positioned outside (TP1) and in the treated region (TP2). The orange rectangle indicates the thermal damage zone.

Two electrodes with an active tip length of 1.5 cm were inserted in the pancreas head parallel to the SMV, as shown in Figure A6A. Two consecutive IRE sessions were performed with an interval of 1 min between the start of the sessions. The pulse parameters are provided in Table 12. The T_TP,init_, T_TP,max_, and ΔT_TP_ of each session are shown in Figure A6B. This figure reveals that T_TP,max_ in the ablation zone ranged from 35.4 °C to 38.8 °C, with ΔT_TP_ ranging from -0.1 °C to 3.2 °C. The ΔT_Treat,max_ was 3.4 °C. This case did not entail thermal camera measurements.

**Table 12:** Pulse parameters and electrode pair (lateral configuration) of the IRE sessions in the pancreas head with superior mesenteric vein in the treated region (case 11).

Session	Electric field strength [V/cm]	Pulse duration [μs]	Number of pulses	Electrode pair
Session 1	1,500	90	10	1-2
Session 2	1,350	90	80	1-2

#### 3.3.5. Case 12: Pancreas head – two electrodes in the vicinity of a constricted superior mesenteric vein

**Figure A7:**
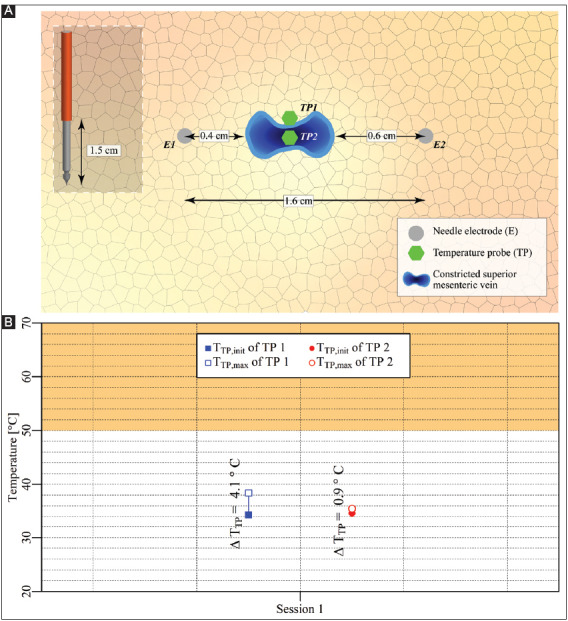
**(A)** Configuration of electrodes and the temperature probes in the pancreas head. **(B)** Measured T_TP,init_, T_TP,max_, and ΔT_TP_ of each temperature probe positioned outside (TP1) and in the treated region (TP2). The orange rectangle indicates the thermal damage zone.

After completing the IRE sessions in case 11, the superior mesenteric vein was constricted to cease blood flow, as shown in Figure A7A. Here, the configurations of the electrodes and the temperature probes were kept the same as in case 11. A single IRE session was performed with the pulse parameters provided in Table 13.

**Table 13:** Pulse parameters and electrode pair (lateral configuration) of the IRE session in the pancreas head with constricted superior mesenteric vein in the treated region (case 12).

Session	Electric field strength [V/cm]	Pulse duration [μs]	Number of pulses	Electrode pair
Session 1	1,350	90	90	1-2

The T_TP,init_, T_TP,max_, and ΔT_TP_ of each probe for each respective session are shown in Figure A7B. This figure reveals that T_TP,max_ in the ablation zone ranged from 34.3 °C to 38.4 °C, with ΔT_TP_ ranging from 0.9 °C to 4.1 °C. The ΔT_Treat,max_ was 4.1 °C. This case did not entail thermal camera measurements.

### 3.4. Correlation between surface temperature and temperature within the treated region

**Figure 8:**
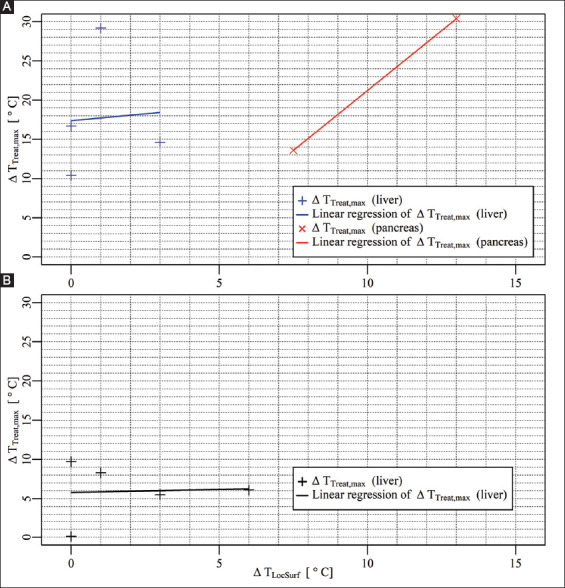
**(A)** ΔT_LocSurf_ versus ΔT_Treat,max_ inside the treated volumes in the liver (+) and the pancreas (×), including the linear regression models. **(B)** ΔT_LocSurf_ versus ΔT_Treat,max_ outside the treated volumes in the liver (+), including the linear regression model.

Figure 8 depicts the correlation between the ΔT_LocSurf_ and the ΔT_Treat,max_ inside the treated region in the liver and the pancreas (Figure 8A) and outside the treated region in the liver (Figure 8B). Inside the treated volume in the liver, the ΔT_LocSurf_ ranged from 0 °C to 3 °C, with ΔT_Treat,max_ ranging from 10.4 °C to 29.2 °C. Outside the treated volume, the ΔT_LocSurf_ ranged from 0 °C to 6 °C, with ΔT_Treat,max_ ranging from 0.1 °C to 9.7 °C. Inside the treated volume in the pancreas, the ΔT_LocSurf_ ranged from 7.5 °C to 13 °C, with ΔT_Treat,max_ ranging from 13.6 °C to 30.4 °C. The linear regression models in Figure 8A were calculated using ΔT_Treat,max_ = 0.35⋅ΔT_LocSurf_ + 17.38 for the liver, and ΔT_Treat,max_ = 3.055⋅ΔT_LocSurf_-9.309 for the pancreas. In Figure 8B, the linear regression model was calculated using ΔT_Treat,max_ = 0.07692⋅ΔT_LocSurf_ + 5.78615 in the liver.

## 4. Discussion

### 4.1. Research questions

#### 4.1.1. Temperature increase depends on the organ and IRE settings

This *in vivo* pilot study demonstrated that IRE is capable of producing sufficient ΔT_Session,max_ to cause thermal damage (T ≥ 50 °C, ΔT_Session,max_ ≥ 13 °C). This depends not only on tissue type and applied treatment parameters but also possibly on the occurrence of electrical arcing. For example, the dependence on tissue type was shown in the liver periphery and pancreatic tail, where the ΔT_Session,max_ in the liver periphery was 18.4 °C higher compared to the pancreatic tail (case 2, session 1, TP1 vs. case 8, session 5). For the applied treatment parameters, a higher ΔT_Session,max_ was observed with an increase in the number of electrode pairs (case 1, session 2 vs. case 2), the electric field strength (case 9, session1 vs. session 2 vs. session 3), and the pulse number (case 9, session 3 vs. session 4). These results are in accordance with the previous studies performed by Dunki-Jacobs et al. and Faroj *et al*. [17,19], who also demonstrated that IRE can cause thermal damage depending on the tissue type and the applied treatment parameters. In these pig studies IRE treatments were applied using several pulse parameters in the liver and the pancreas, while measuring the temperatures in the treated volume. For instance, Dunki-Jacobs *et al*. demonstrated higher ΔT_Session,max_ in the liver than in the pancreas, while Faroj *et al*. showed higher ΔT_Session,max_ for increasing electric field strength and pulse number in the liver.

Despite the significant influence of the applied treatment parameters on ΔT_Session,max_, a recent *ex vivo* study by O’Brien *et al*. concluded that it is still possible to reduce the ΔT_Session,max_ by choosing an adequate order of the electrode pairs, and by applying a cycled pulsing scheme where the total number of pulses per electrode pair is subdivided and repeated in several cycles [22]. Using a perfused porcine liver model that cycled pulsing schemes it was possible to reduce the electrical current, increase the treatment zone size, and ultimately maintain a low tissue temperature compared to conventional pulsing schemes. Future *in vivo* studies should therefore investigate the thermal increase when cycled pulsing regimens are used.

Another element that can cause thermal damage is electrical arcing. In this phenomenon, an instantaneous increase in the electrical current density develops between the activated electrodes, resulting in (1) possibly inadequate treatment due to the redistribution of the electric field [23], and (2) significant temperature increase that can cause, for example, white coagulation [18,24,25]. During the IRE treatment, the electrical arcing is experienced as audible popping sound with visible electrical current spikes and possible system failure [23,25]. This phenomenon likely occurs because of:

- High or increase in the electrical conductivity of the IRE-subjected tissue volume between the activated electrodes [25];
- Ionization of gases that were formed by electrolysis of water into oxygen (O_2_) and hydrogen (H_2_) due to large electric field strengths[20,26,27].

To prevent this anomaly and reduce the probability of thermal damage, physicians are recommended to reduce the pulse voltage or the pulse width [5,23,25].

#### 4.1.2. Surface temperature misrepresents ablation zone temperature

Temperature elevations induced by IRE in the treated regions sometimes produce a substantial rise in surface temperature. Our findings generally revealed a minimal temperature increase at the liver’s surface (with a median of 1 °C) and a relatively large temperature increase at the surface of the pancreas (with a median of 10 °C). This could be explained by the positions of the active electrodes with respect to the surface of the organs. Specifically, the active electrodes could be situated far from the hepatic surface, and close to the surface of the pancreatic tail. Another explanation could be higher perfusion in the liver, resulting in faster heat removal (heat sink effect) and therefore a smaller temperature rise at the surface of the liver.

In the clinic, it is not always feasible to perioperatively measure the surface temperature of the organ, even though it seems attractive as a simpler, time-saving alternative for temperature measurements with invasive probes. Such impracticality particularly holds when IRE treatments are performed percutaneously. For open procedures, the surface temperature may not significantly increase or even correlate with the maximum temperature increase during/after the treatment, despite the weak positive linear correlation, as was shown here for the liver. In our study, clinical pulse parameters were applied and the findings are hence translatable to the patient setting. In line with the findings, we recommend physicians to use invasive IRE-compatible temperature probes (probes that do not disturb the electric field distribution, e.g. non-metal probes) instead of surface measurements to accurately monitor perioperative temperature build-up.

#### 4.1.3. Moderate hyperthermia in tumors may confer adjuvant effect on therapeutic efficacy

Although excessive temperatures can cause severe complications such as biliary obstruction and stenosis of blood vessels [2], moderate heating may enhance the IRE effect [28]. Edelblute *et al*. demonstrated that heating at 43 °C for 1-2 min significantly enhanced *ex vivo* IRE tumor ablation of Pan02 xenografts by 5.7-fold at 750 V/cm and by 1.7-fold at 1,500 V/cm. Furthermore, heating of the tumor area in an ectopic mouse model to 42 °C during IRE treatment almost doubled the median survival [16]. Pre-heating the tumor reduces the impedance of the tumor [29], thereby decreasing the IRE threshold. Accordingly, local heat evolution should be considered a welcomed side effect of IRE as long as it does not exceed mild hyperthermia that remains confined to the IRE ablation zone.

#### 4.1.4. Heat sink effects are minimal in the liver and pancreas

Unexpectedly, the heat sink effects caused by the portal vein in the liver and the superior mesenteric vein in the pancreas were minimal. For example, after application of IRE in the vicinity of the portal vein in the liver hilum (case 3), a temperature increase of almost 6 °C was measured in the bile duct outside yet proximal to the treated region. Because of the poor contact of the temperature probe with the wall of the bile duct, this 6 °C can be considered an underestimation and hence possibly detrimental to such vital structures. Furthermore, in the pancreatic head with the superior mesenteric vein located inside the treated region (case 11), the ΔT_Session,max_ was only 3 °C higher compared to that in case 10, where the superior mesenteric vein was located near the treated region.

There might be a (large) variation in blood flow and electrical resistance between the different animals used in the different experiments, possibly explaining the variation in ΔT_Session,max_. Furthermore, the flow resistance that was caused by the placement of the temperature probe might also affect the local blood flow. However, the most plausible explanation for the minimal heat sink effects is reduced blood flow due to anesthesia. This is illustrated in case 12, where the superior mesenteric vein was constricted, though only a 0.9 °C higher ΔT_Session,max_ was registered compared to case 11. Both experiments were conducted in the same animal and a relatively low blood flow due to anesthesia could explain this relatively small difference. Further investigations should be performed to confirm whether the anesthesia adversely affects the heat sink effects caused by large blood vessels, including the superior mesenteric artery and the hepatic artery. In addition, the relatively short exposure to IRE could also contribute to the observed effect that sometimes the temperature increase was limited near large vessels.

#### 4.1.5. Metal stents amplify the extent of heating

The presence and the orientation of the metal stent in the bile duct influence the ΔT_Session,max_. For instance, the experiments that included two electrodes parallel to the bile duct (case 6 and case 7) revealed that the presence of the stent resulted in a higher ΔT_Session,max_ of 19.8 °C compared to similar IRE procedures in the absence of a stent. The experiments with three electrodes showed that the parallel orientation of the stent (case 4) resulted in a higher ΔT_Session,max_ of 4.2 °C compared with perpendicular orientation (case 5).

These results are in line with the porcine study performed by Dunki-Jacobs *et al*. and with the phantom study performed by Scheffer *et al*. [17,30]. However, Scheffer *et al*. reported a higher ΔT_Session,max_ in the phantom study when the stent was perpendicular to the electrodes, while we found a higher ΔT_Session,max_ for a parallel orientation. This can be explained by the fact that, in the phantom composed of homogeneous tissue-mimetic material, the perpendicularly placed stent logically resulted in reduction of the length of the electrical current paths, resulting in larger electric current near the stent and, therefore, a greater increase in temperature. In our *in vivo* study, the stent was placed in the bile duct that has a wall with a comparatively large electrical resistance. Since the bile duct interrupts the current paths of the electrode pairs 1-2 and 3-1 in case 5, where electrodes were placed perpendicular to the bile duct, the length of the current paths was extended, resulting in larger electrical resistance (which means less electrical current) and, therefore, a lower ΔT_Session,max_.

According to case 4 through 7, placement of electrodes near a metal stent possibly produces thermal damage, also in surrounding normal tissue, which may explain the complications reported by Mansson *et al*. after applying IRE in the vicinity of a metal stent [31]. Thus, caution is warranted when a metal stent is placed in the treatment region and further investigations are required to determine the influence of the stent on the exacerbated ΔT_Session,max_ that can be expected in the treated region.

### 4.2. Study limitations

This study has some limitations. First, the ΔT_Session,max_ is underestimated in cases with a single electrode pair because the temperature increments in these cases are the highest near the electrode. Moreover, IRE was performed using an open surgery approach. It could be argued that, inasmuch as the operating room ventilation system was turned on, the surface temperature of the organs was lowered, which may have widened the temperature difference between the surface and tissue. Environmental cooling, however, is also employed in the human operating room setting and was therefore appropriately implemented in the experimental design. Another possible limitation is the application of IRE to healthy tissue. Several studies have found that healthy tissue has lower electrical conductivity than cancerous tissue [32,33]. This implies that ΔT_Session,max_ in the treated volumes are underestimated in our study. However, Beitel-White *et al*. showed that healthy human hepatic and pancreatic tissues have higher electrical conductivities than the cancerous human hepatic and pancreatic tissue after the application of IRE [34], which validates the application of IRE in healthy tissue in this study. Still, limited data are available on electrical conductivity of IRE-subjected tissues. Finally, results are obtained from a case study series and a larger number of experiments would allow more robust conclusions. Nevertheless, the study provides very relevant insights that can contribute to future improvements in IRE protocols and make clinicians employing the technique aware of its limitations and factors that exact caution.

### 4.3. Future directions: Numerical treatment planning

The differences in organ-specific properties and their practical implications give rise to the notion that, in the future, numerical treatment planning should be performed to optimize IRE treatment. In the envisaged treatment planning protocol, numerical calculations are performed on 3D computer models constructed from patient-specific medical images before the IRE treatment. For each patient, parameters of pulses (such as voltage, duration, frequency, and number) and electrodes (such as configuration, number, active length, location, and distance between electrodes) are numerically optimized. This is done by calculating the distribution of the electric field strength and temperature in the treated volume, ensuring the coverage of the entire target volume with sufficient electric fields to produce a membrane permeabilization effect while reducing the probability of the thermal damage. In the 3D computer model, it is important to include perfusion effects to account for thermal redistribution in the treated volume, and large blood vessels to consider the heterogeneity of the tissues and to avoid a possible “electric field sink” effects [35,36]. The electric field sink is the reduction of the electric field strength in the target volume due to the presence of blood vessels in or near the target volume, which could result in incomplete treatment. The electric field sink effect depends for instance on the orientation of the electrode configuration with respect to the blood vessel and the diameter of the blood vessels [35].

Furthermore, the inclusion of large blood vessels can also be used to account for the local heat sink effect in regard to the thermal ablation techniques, such as radiofrequency ablation (RFA) and microwave ablation (MWA). Large blood vessels can yield a substantial local cooling effect in the treated volume, as was shown by several experimental and *in silico* studies [37-40]. However, to the best of our knowledge, no *in silico* study has considered the possible reduction of blood flow presumably due to anesthesia, which results in a possible underestimation of temperature increase. Therefore, the blood perfusion rate under the anesthesia effect should be investigated for more accurate treatment planning.

## 5. Conclusions

Based on this pilot study in porcine liver and pancreas, we conclude that IRE can produce sufficient temperature elevation to cause thermal damage, depending on tissue type, perfusion status, and IRE parameters such as the number of electrode pair, the electric field strength, and pulse number. The temperature elevation can be further influenced by the presence and orientation of a metal stent in the treatment zone. In the clinic, a temperature above 50 °C for a certain duration may undesirably damage vital structures proximal to the target tissue and cause clinical complications. Furthermore, the temperature elevation in the treatment zone is not always reflected in the surface temperature. Perioperative surface temperature monitoring therefore has little clinical value. Finally, the heat sink effect caused by the portal vein in the liver and the superior mesenteric vein in the pancreas is minimal. In the future, numerical treatment planning can be considered to optimize the IRE treatment and minimize thermal damage.

## Potential Conflicts of Interest and Sources of Funding

This research was supported by the Dutch Cancer Society (Grants no. 2014-7244 and 10666). The author(s) declared the following potential conflicts of interest with respect to the research, authorship, and/or publication of this article: K. P. van Lienden, T. M. van Gulik and M.R. Meijerink are paid consultants for AngioDynamics. AngioDynamics had no role in the design of the study; in the collection, analyses, or interpretation of data; in the writing of the manuscript, or in the decision to publish the results.
